# Anatomist Hermann Stieve used Nazi victims for his research on the menstrual cycle, but did he also fabricate facts?

**DOI:** 10.1007/s00404-022-06664-4

**Published:** 2022-08-08

**Authors:** Andreas Winkelmann, Isabel Freiberger

**Affiliations:** 1 Institute of Anatomy, Brandenburg Medical School, Fehrbelliner Str. 38, 16816 Neuruppin, Germany; 2 Faculty of Health Sciences Brandenburg, Brandenburg Medical School, Potsdam, Germany

**Keywords:** Menstrual cycle, Histological research, National Socialism, Capital punishment

## Abstract

**Purpose:**

Hermann Stieve (1886–1952), director of the Berlin Anatomical Institute from 1935, benefited from the rise of execution numbers during the “Third Reich”. He used organs and tissues from executed women for his histological research on the reproductive organs and investigated the influence of “nervous agitation” on the cyclical changes of endometrium and ovary. It is still controversial how he was able to acquire intimate data on the executed women and it was therefore suggested that some of his data may have been “invented”.

**Methods:**

Newly emerged dissection protocols and histological drawings from Stieve’s research, together with archived court records, enable a more detailed analysis of Stieve’s published data.

**Results:**

We extracted 304 case descriptions from Stieve’s publications. Of these, 88 could be linked with 33 identifiable women and related historical records. Nearly all reported causes of death and/or verdicts of executed women were false. Reported clinical data, particularly the day of the menstrual cycle and uterine bleeding shortly before death, are more difficult to verify. We found non-standardised documentation and possible confusions of cases, which may in part be attributable to war effects.

**Conclusion:**

Stieve actively concealed the fate of the executed women, mostly by inventing imaginary stories. This followed a request by the German and Soviet authorities after 1945 not to publish results from cases of political victims, but only from “dangerous criminals”. Scientifically relevant clinical data were not always reported correctly, but are not necessarily fraudulent as different interpretations of this finding can be suggested.

**Supplementary Information:**

The online version contains supplementary material available at 10.1007/s00404-022-06664-4.

## What does this study add to the clinical work

**Table:** 

This historical paper shows that scientific understanding of the menstrual cycle is a rather recent achievement. It reminds clinicians that histology only offers a frozen moment in time and that the relationship between clinical and histological findings is intricate.	

## Introduction

Hermann Stieve (1886–1952) was anatomist in Leipzig, Halle/Saale and, from 1935 in Berlin [1]. His main research interest throughout his career was the histology and function of the reproductive organs. It is well known by now that for this research, Stieve had access to the bodies of execution victims. Stieve organised that most bodies of those executed at the Berlin Plötzensee prison under the Nazi regime were used for research or teaching at his institute [2–7]. It is also known that he used the stressful situation of women on death row for his research to study the influence of the nervous system on the morphology of ovary and uterus. Even if anatomists traditionally had access to bodies of the executed and Stieve was not responsible for the situation of these women, it remains difficult to accept that he benefited from it in this way.

Even after the war, bodies of executed individuals were given to the anatomical institute as the new allied authorities did not see this practice as Nazi-typical [8]. Nevertheless, Stieve had to justify his use of these bodies, particularly in the case of *political* victims, before the university administration, but also before the scientific community [9, 10]. The administration and the Soviet authorities only allowed Stieve to continue publication of his research if the studied women remained anonymous and either the subjects were “truly criminal” or the identity of political victims remained covert [3, p.85]. Accordingly, Stieve continued to publish his research results, but actively concealed or obscured the identity and true fate of the women whose tissues he had investigated [8]. As he had never joined the NSDAP and—as so many others—had successfully suggested that he had even offered resistance to the regime (which he had not), his professorship remained uncontested and he died as a respected anatomist and researcher with a good reputation well into the 1980s as the “anatomist of the gynaecologists” [11].

Stieve earned this “title” as his research—published in anatomical and gynaecological journals including the *Archiv für Gynäkologie*—was directly relevant to contemporary scientific debates regarding the menstrual cycle. Stieve’s main scientific hypothesis was that the genital organs stand under direct influence of the nervous system, explaining the effects of “nervous agitation” and massive fear on these organs [12]. According to this hypothesis, nervous influence can lead to atresia of follicles in the ovary and to amenorrhoea, and the direct influence on the uterus can produce what Stieve termed “*Schreckblutung* [shock bleeding]”. He also postulated “paracyclical” ovulations, i.e., ovulations at any time of the menstrual cycle, which he hypothetically attributed to hyperaemia of the pelvic organs triggered by nervous stress or sexual arousal. This brought him into opposition to Austrian gynaecologist Hermann Knaus and his suggestion that infertile periods were predictable [13, 14].

Stieve was a morphologist and his method was classical histology, judging tissues by visual inspection through the microscope. Stieve’s interest also included the function of these tissues, and one feature of his research was correlation of morphology with clinical findings, particularly with events of the menstrual cycle. This linked him to gynaecological histopathologists like Robert Schröder or Robert Meyer, and it also led him to cooperation with gynaecologists in Halle and Berlin, who provided surgically removed organs and clinical information. When the NS (National Socialist) justice system started to extend capital punishment from 1933 onwards, now including more and more women, this was an (obviously macabre) alternative for Stieve to obtain tissues from young women. From 1935, Stieve therefore actively cooperated with the justice system to facilitate his research, with access to the bodies usually within one hour after death and with the obvious stresses of death row as a research topic.

A hallmark of Stieve’s publications are his elaborate case descriptions that bring the individual histology of ovary and uterus close to the women’s biography, including not only details of their cause of death and/or (alleged) crimes, but also intimate detail of their sexual life and their menstrual cycle. It is still controversial how Stieve gained access to such information. After the war, he explained that his sources had been court records, prison doctors and warders, or even the families of the deceased. All these sources can, however, be doubted from today’s point of view [3]. If, however, Stieve did not have access to this information, the question emerges whether he manipulated or even “invented” data, as Tuchel has recently suggested—while also conceding that there is no evidence for this assumption [3].

Investigation of this question has so far been hampered by the lack of historical documentation of Stieve’s research and the unexplained post-war disappearance of the body register of Stieve’s institute. The only surviving research document was a list of 174 women and 8 men, all Plötzensee victims, which Stieve handed over to the administration in 1946 [15]. However, this list is clearly incomplete [16] and cannot be linked to individual case descriptions. The recent emergence of estate material of two of Stieve’s sons offers new sources to investigate this question. This material, described in detail below, includes names and links some of Stieve’s case descriptions to individual Plötzensee victims. It thus offers an opportunity to fact-check the detailed clinical-biographical information Stieve gives in his publications.

## Sources and methods

The estate material handed down through two of Stieve’s sons is now at the archive of Humboldt University in Berlin under “HU UA, NL Stieve” (subunits have no signatures of their own yet). The records preserved by the first son Friedrich–Ernst Stieve (1915–2012) did also include histological slides and have already been described on the occasion of their burial [16]. They consisted of the following:

- 278 histological slides, of which 106 included non-human tissue. 36 slides carried inscriptions with a total of 20 names, of which 15 could be unequivocally identified as men and women executed at Plötzensee prison. The 172 slides with human tissue were buried in Berlin in May 2019 [16].
- 990 histological drawings, mostly in black ink on cardboard, all produced by Munich-based artist Berta Neresheimer (1878–1971). On their reverse, most drawings hold inscriptions, mostly in Stieve’s own hand and some including shorthand. The drawings will be referred to as "Neresheimer drawings" by their consecutive numbers 1–990 added for archiving.
- Offprints of all of Stieve’s publications (around 250).
- 143 letters and documents, mostly personal correspondence and certificates. These did not include much new information relevant to this investigation.

Material preserved by Stieve’s second son Robert Stieve (1922–1981) emerged more recently and has already been described elsewhere [8]. It mainly included more than 200 dissection protocols in Stieve’s own hand from between 1942 and 1947, 206 of them referring to named executed women. On these protocols, Stieve had noted basic information like age and date of death, his dissection findings focussed on the genital organs, mostly also the length of imprisonment, and occasionally the reason for the verdict. About one third of protocols included histological findings. The protocol sheets gave the impression that they were regularly used as a basis for publications [8]. These dissection protocols will be referred to by their original consecutive numbers.

Case descriptions were extracted from Stieve’s publications with the aim of linking them with historical individuals. They were tabulated including basic information like age of the described individual, occasionally given initials, cause of death, number of children, information on the menstrual cycle, and figures related to the case. To make the number of case descriptions manageable, the investigation was restricted to publications on the female reproductive organs and to three representative time periods: 1927–1930, 1942–1943, and 1950–1952. These represent three different phases of Stieve’s research: first, a time when Stieve was still in Halle and had no access to executed women; second, years during the war, when execution numbers were very high and Stieve, now in Berlin, could investigate tissues and organs of many younger women; and third, post-war years, when Stieve would continue to work on the “material” he had gathered in the years before [8]. The specific years were chosen to study comparable numbers of case descriptions for the three periods.

Individual names appearing on the histological slides have already been published [16]. All inscriptions on the drawings were transcribed and those with names extracted. Names and information from the dissection protocols were also tabulated. By comparing published figures with drawings including names and case characteristics with information on the dissection protocols, we tried to link published cases with names.

Names that could be linked with cases with sufficient certainty were followed up historically using records, mostly from the prison or court administration, at archives in Berlin and Brandenburg and at the *Gedenkstätte Deutscher Widerstand* (GDW, German Resistance Memorial Center) with the aim of identifying as many historical individuals as possible and recording biographical information. This was necessary to compare Stieve’s descriptions with independent historical records. As numbers generated by our survey of cases are often based on subjective interpretation, which sometimes remained ambiguous, we refrained from statistical analysis, so that all comparisons made remain descriptive. All translations from German to English are our own.

While there have been calls to publish all names of NS victims who have been subject of medical research during NS times [17], we find it debatable to link specific dissection findings and descriptions of organs and/or of details of the menstrual cycle with individual names. While it is important to remember NS crimes and to commemorate the victims, we doubt that the anatomical description of individual organs should be part of that commemoration. Moreover, such an approach would risk to repeat the objectification of the victims inherent in Stieve’s research approach. However, to link Stieve’s published case descriptions with identifiable women and related historical sources was our only chance to fact-check Stieve’s published data. We therefore have to refer to specific historical sources to make our investigation reproducible. We nevertheless abstain from openly presenting names in the context of individual case descriptions. For individual commemoration, information on all victims of Plötzensee executions can be retrieved from documentation at the GDW in Berlin. Names of those women who appear in Stieve’s records (virtually all women executed in Plötzensee) have been published previously [8, 15, 16].

## Results

About half of the 990 Neresheimer drawings can be related to images—and mostly also case descriptions of various length—in Stieve’s publications spanning years from 1913 to 1952. The inscriptions on drawings included a total of 52 names (on 119 drawings) that were neither names of gynaecologists or anatomists providing material (like Stoeckel or Sellheim) nor eponymous names of histological techniques (like Zenker), but most likely names of individuals whose tissues were depicted. 41 of these 52 names could be related to historical persons. This actually includes Stieve’s own wife, whose palatine tonsil was among the drawn organs. All other identifiable individuals were women executed either in Plötzensee or, in one case of after 1945, in another Berlin prison. The remaining 11 names include names difficult to read and three names that according to Stieve were from a murder victim, a woman who died of starvation, and a case of surgical removal of organs, respectively.

On the histological slides [16], eight additional names appeared that were not in the drawings (of three men and five women), but as these could not be related to any case descriptions, they will not be considered further here.

### Case descriptions

Stieve based most of his scientific conclusions on the presentation of individual cases or case series. We present here an exemplary case description that was first published in 1943 [18] and, with virtually identical wording, as case “14L” in 1952 [12 (p. 76)]:

“*Es handelt sich um eine 31 Jahre alte, unverheiratete Frau, die angeblich immer gesund war. […] Seit dem 14. Lebensjahre hatte sie alle 26–34 Tage menstruiert, die Blutung währte 4–5 Tage und verursachte keine nennenswerten Beschwerden. Die Frau war zweimal verlobt und hatte seit dem 22. Jahre regelmäßig geschlechtlich verkehrt, eine Schwangerschaft war stets verhindert worden. Die Frau machte einen ruhigen, wenig begabten Eindruck. Sie arbeitete fleißig und zuverlässig. Wegen eines schweren Verbrechens kam sie ins Gefängnis. Daraufhin blieb die Blutung zunächst aus […]. [A]m 128. Tage nach der Einlieferung ins Gefängnis, erhielt die Frau eine Nachricht, die sie sehr stark erregte. Kaum 1 Stunde später trat eine schwache Blutung aus den Geschlechtsorganen auf, die die Frau für eine Menstruation hielt. 8 Stunden später konnte ich folgenden Befund erheben*:

*Es handelt sich um eine kleine, zierlich gebaute Frau mit zartem Knochengerüst, gut ausgebildeter Muskulatur und gut entwickeltem Fettpolster. …*”.

Translation: “It is a 31-year-old unmarried woman who allegedly has always been healthy. […] From age 14 she had menstruated every 26–34 days, the bleeding took 4–5 days and did not cause significant discomfort. The woman was engaged twice and had regular sexual intercourse since age 22, pregnancy had always been prevented. The woman appeared calm and less able. She worked diligently and reliably. She was imprisoned for a felony. Subsequently, the [menstrual] bleeding stopped […]. On the 128th day of her imprisonment, the woman received a message that agitated her massively. Less than an hour later, a weak bleeding from the sexual organs occurred, which the woman took for a menstruation. 8 h later, I was able to gather the following findings:

It is a small, slender woman with a delicate ossuous framework, well-built musculature and well developed fat pad. …”.

This case description is typical in that Stieve describes many aspects of the woman’s sexual life and biographical details including stressful events, and in the next paragraph seamlessly reports the bodily findings of his dissection. These are then followed by histological description of the sexual organs, usually accompanied by figures drawn by Neresheimer.

We extracted a total of 304 case descriptions from Stieve’s publications. The number for each of the three defined time periods and the provenance of the specimens as reported by Stieve are listed in Table 1. It is evident from the numbers that Stieve’s sources for tissue changed dramatically from before the war, when most of his specimens (92%) originated from surgery or from death by natural causes, to during and after the war, when the sources are either not disclosed, include more deaths from non-natural causes or, in more than 40%, likely stem from executed prisoners (cf. Table 1). As we will show below, this information is not always correct and includes even more executed women.

**Table 1:** Characteristics of case descriptions in Stieve's publications

	1927–1930	1942–1943	1950–1952
*Identified case descriptions*	*133*	*81*	*90*
**No clear information**	12 (9%)	5 (6%)	21 (23%)
**Surgical and embryological specimens**			
**Total**	**90 (68%)**	**31 (38%)**	**10 (11%)**
Surgical specimens	19 (14%)	8 (10%)	10 (11%)
Embryological specimens	71 (53%)	23 (28%)	–
**Death not suggestive of execution**			
**Total**	**30 (23%)**	**9 (11%)**	**21 (23%)**
Death by natural causes	21 (16%)	–	–
Death by starving	–	–	1 (1%)
Suicide	4 (3%)	1 (1%)	4 (4%)
Death by accident or “﻿outer forces”	3 (2%)	8 (10%)	5 (6%)
Death by air raid	–	–	9 (10%)
Death by murder	2 (2%)	–	2 (2%)
**Death suggestive of execution**			
**Total**	**1 (1%)**	**36 (44%)**	**38 (42%)**
Imprisonment	–	15 (18%)	19 (21%)
“Sudden death”, without further details	1 (1%)	5 (6%)	4 (4%)
“Perfectly healthy”, without further details	–	3 (4%)	4 (4%)
Information directly suggestive of execution	–	13 (16%)	9 (10%)
Execution is reported	–	–	2 (2%)

Case descriptions of the three investigated time periods. Several case descriptions often refer to the same woman, but may nevertheless include divergent information

For 88 of these case descriptions (29%), the historical person behind the case could be identified, none of them in publications of 1927–1930. Identification was possible when drawings with an inscribed name matched the published images and when age and initial published by Stieve matched the person. In 27 of 33 cases, the identification was confirmed by the dissection protocol, which was also compared with published information. For the remaining six, no dissection protocol was available. Except in one case, surgically removed organs that Stieve often used for his research were not linked with individual names. These cases are therefore difficult to follow up historically. Several individuals were used for publication more than once. In total, 33 identifiable women could be related to one or more of the published cases, which will form the basis of the following analysis. 32 of these women were executed in Berlin Plötzensee between 1935 and 1945, and one woman was executed in another Berlin prison in 1947.

### Causes of death and/or verdicts

The information Stieve gives in individual cases regarding the cause of death and, in the case of executions, the verdict, was easily verified historically as archival records with relevant information were available for all 33 identified individuals, see the list below. In cases related to the same women but published more than once, the publication with the most detailed information was considered:

**Table:** 

No information	8
Vague information (“died suddenly”)	7
Reports false cause of death	
Air raid	4
Suicide	2
Reports execution for	
“Felony”	5
False verdict / Murder	4
False verdicht / Other	1
Correct verdict (murder)	2

The results confirm that Stieve does not necessarily conceal the fact that the women whose organs he investigated were executed, but in many individual cases he either remains vague or even reports false causes of death, and in most cases he does not give the true reason for the women’s death sentence.

In 14 of the 32 cases of women executed in Plötzensee, Stieve either gives no information regarding the cause of death or vague information like “died suddenly”. In six cases, Stieve obviously invents false causes of death, either air raids or suicide. In ten cases, he indicates execution as the cause of death, but reports verdicts rather vaguely as “*schwere Verbrechen* [felony]” without further explanation, or simply reports “murder” as the reason of the verdict in cases of women who were not accused of murder. This includes historical cases of (alleged) treason as well as cases of petty crimes, which could nevertheless attract capital punishment under special NS laws (see below).

Only in two cases of capital punishment for murder, Stieve reports the correct verdict. In one of them, of 1942, Stieve sees no need to conceal the verdict as an executed murderer was not seen as a political victim. In the second case, the only execution of after 1945 among these 33 cases, he additionally refers to the support of the allied authorities for the court decision (cf. [8] for more detail).

In one case, Stieve tells an elaborate story of a woman who allegedly killed seven men by poisoning [12, p.49], while the 35-year-old woman whose organs he describes was actually convicted of plundering and executed in February 1945.^1^ In this case, Stieve’s story is obviously taken from another case, a 37-year-old woman who did kill seven people by poisoning and was executed in Plötzensee in May 1944.^2^ The latter woman was also dissected by Stieve as is attested by the existence of the dissection protocol. However, this protocol describes different anatomical and histological findings than the case described in [12], so that it seems unlikely that Stieve completely mixed up these two cases. Either he mixed up the biographical stories behind the cases when publishing them, or he intently used the high-profile story of sevenfold murder to conceal the true fate of the woman in question.

One example for the vague and rather incorrect label of felony (“*schweres Verbrechen*”) is the case quoted above. In this case, the case label “14L” together with the inscription of a name on drawing no. 914 of the estate, an image published with the case, allow the identification of this woman as being convicted of stealing valuables from parcels of army soldiers,^3^ a crime that was punishable by death based on the *Volksschädlingsverordnung* (law against antisocial parasites), a NS law established in 1939 to enforce uncritical solidarity with the Reich’s war effort. Stieve uses the same label “*schweres Verbrechen* [felony]” in 1943 [18] and 1952 [12] even though by 1952, it should have been clear that theft, even in war times, did not deserve this label. Whether Stieve himself saw it as a crime that deserved capital punishment in 1943, or even in 1952, is not discernible from the records and must remain speculation.

**Fig. 1:**
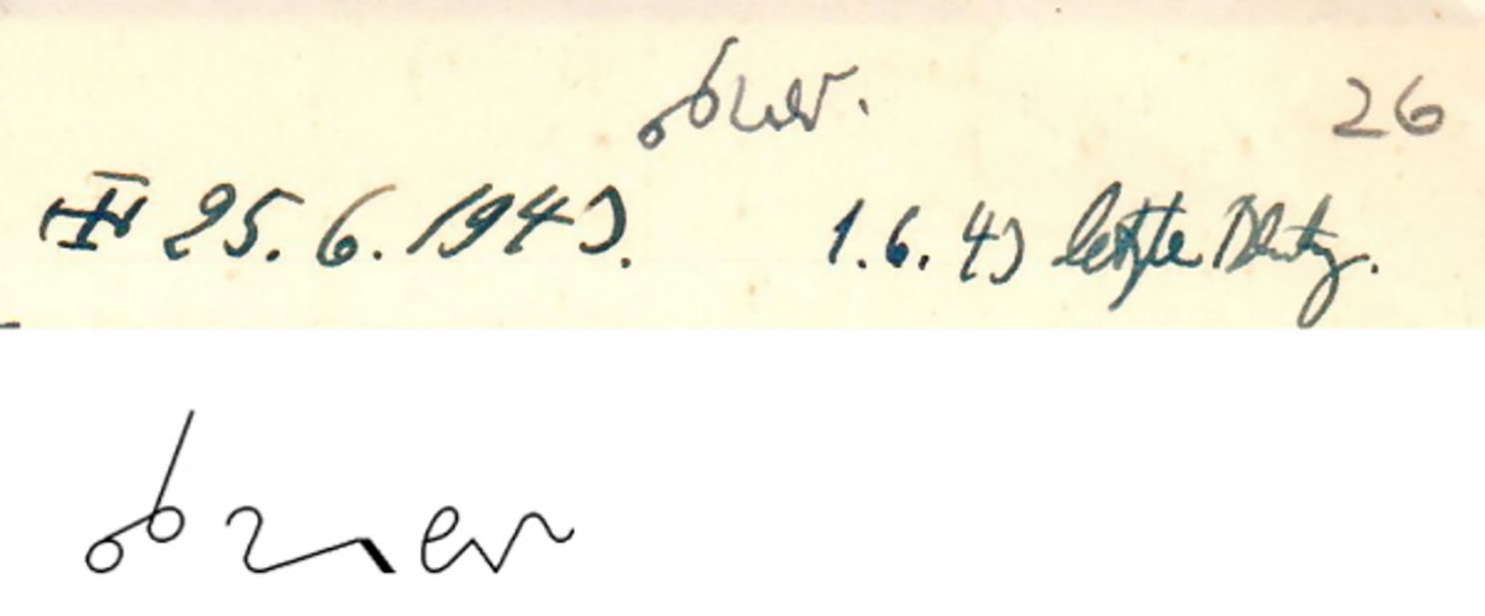
This detail of a dissection protocol documents a false cause of death. Above: detail of the upper right hand corner of dissection protocol no. 26. “*letzte Blutung*” translates as “last bleeding”. Below: transliteration in *Deutscher Einheits-Kurzschrift* (German Standard Shorthand) available at http://steno.tu-clausthal.de/DEK.php. for “*sest mör derin* [*Selbstmörderin*]”, i.e., suicidal woman, allowing for an individual style of shorthand that Stieve may have acquired over the years

There are three cases where Stieve adds the *invented* cause of death to the dissection protocol, two cases of suicide and one case of infanticide. As an example, we present one of the cases of suicide: A 25-year-old woman was sentenced to death for espionage and executed in June 1943 [19]. The related dissection protocol (no. 26) includes an addition in shorthand next to the date of death that can be decoded as “Selbstmörderin [suicidal woman]” (Fig. 1). Stieve knew that this woman had been executed and had certainly not committed suicide. Nevertheless, he noted this information on the dissection protocol, perhaps to be consistent in further publications. The case was actually reported identically in two publications, [20, p.124] and [12, p.40].

### Information relevant to Stieve’s scientific arguments

#### Negligent documentation and reporting?

Before looking at individual case descriptions, it must be noted that studying Stieve’s publications and documentation, we came across quite a lot of easily identifiable errors. These can be divided into three groups. The first comprises minor errors, likely oversights, that have no consequence for the scientific findings. For example, in several cases, a published figure depicts the left ovary (Fig. 43 in [12]) while an inscription on the identical drawing (Nos. 914 and 944, respectively) indicates the right ovary.

The second group comprises errors that have an unclear or minor influence on scientific findings, but may point to a certain negligence. This includes the reported age of the women, which in 31 of the 207 dissection protocols is wrong by 1 year, apparently because the year of birth was simply subtracted from the year of death without paying attention to the day and month. In the same vein, many different spellings of names occur, particularly in non-German names [cf. 8]. This may simply be attributed to typos, but may also cast doubt on the identity of cases. For example, drawings carry the names Tyskowski (no. 843), Tyschenowski (849), Tyschewski (960), or Tyschewska (962), which may or may not be identical with the name Pyschewski on Stieve’s list (no. 133 [8]) and with the name of the historical Plötzensee victim Paula Tyschenski (1899–1941). Stieve also makes mistakes in the calculation of the days of the menstrual cycle. In a publication of 1946, Stieve gives the first day of the last menstrual bleeding as 5 December and the day of death as 29 December, but nevertheless speaks of the “24th day” of the cycle [21], repeated identically in 1952 [12, p.53]. Moreover, Stieve usually miscalculates the reported time of amenorrhoea (between 34 and 322 days, see below) by simply equating it with the duration of imprisonment. There is only one case, where he correctly adds the days between the last bleeding and the imprisonment to the days in prison to calculate the total days of amenorrhoea [22, p. 9]. This does not necessarily make a big difference for his scientific argument, but pretends an exactness that is not in the data.

The third group of errors may actually cast doubt on some published results, as they clearly indicate a mix-up of individual cases. There is for example one drawing, no. 938, which carries two different names at the same time, “Jaster 45 years” and “Biesenak 41’”, which may well correspond to two Plötzensee victims Martha Jaster (1899–1944) and Emilie Biesenack (1899–1943). The identical image was, however, published as case “23S” of a 41-year-old woman (R227, p128), who could not be identified but whose last name would start with “S”. Another drawing, no. 940, carries the name Comelli, likely related to Jeanne Comelli (1924–1943), but was published as Fig. 72 related to case “5R” [12, p.134], which designates an identified woman of different age. It remains difficult, however, to quantify and rate such errors.

#### Clinical information

The clinical-biographical information that Stieve reports in his publications and that is often relevant to his scientific argumentation is more difficult to verify or falsify from historical records. When, for example, Stieve states that a woman was executed on the 26th day of her menstrual cycle, this can simply not be fact-checked as no historical records including such information have been found. Nevertheless, some reported clinical-biographical details can be checked against historical sources. This includes the number of pregnancies or children, sometimes used by Stieve to indicate a normal sexual function, and the duration of imprisonment. The latter was important as a possible source of stress (“nervous agitation” in Stieve’s words) and a plausible cause of amenorrhoea and was therefore relevant to Stieve’s research. We therefore analysed this kind of information for the 33 cases of identified execution victims. In 4 out of 25 cases, the given number of pregnancies and/or children was verifiably wrong, and in 4 out of 19 cases, the reported period of imprisonment was clearly different from the one given in the historical records.

We then looked at all 33 cases individually to compare information given in Stieve’s publications, the context of Stieve’s scientific argument in these cases, and historically verifiable information. As neither the case descriptions nor the historical sources are standardised, it is difficult to label case descriptions, or parts of them, simply as ‘true’ or ‘false’. In seven cases, either clinical information in the case description or historical information was minimal, so that data could not be meaningfully compared. In most other cases, there were minor incongruences but no obvious mismatch with a demonstrable effect on scientific conclusions could be detected. However, in a total of nine cases, we found constellations that suggest that scientifically relevant aspects of published clinical-biographical information are at odds with information from historical records regarding the identified women. These nine cases will be summarised in the following. A more detailed discussion of every case, that allows to follow the complexities of the cases and the difficulties of “fact-checking”, is given in the supplementary material.

In four of these cases (cases 1–4, see supplementary material), Stieve gives the number of two to four children in women who, according to the historical records, did not have any children. In all of these four cases, the case description and the related scientific argument suggest that Stieve may have intended to give an impression of a healthy woman of normal reproductive capability by reporting previous childbirths. In two cases, this underpins the presentation of images of *normal* follicles and oocytes (cases 1–2), in one case, it supports the idea of “paracyclical” ovulations in an otherwise healthy and normal genital system (case 3), and in one case, it underpins a report on previously healthy and “stable” ovaries which nevertheless change dramatically under the influence of the nervous system (case 4). In two of these four cases (cases 1 and 3), notes on the dissection protocol suggest that Stieve definitely knew that these women had no children.

In five other cases, Stieve correlates a certain sequence of stressful biographical events with certain events in the menstrual cycle that he reconstructs from his histological findings, for example by judging the age of a corpus luteum to estimate the day of the last ovulation or by predicting an imminent follicle rupture based on the size and appearance of a follicle. However, the reported sequence of biographical events is at odds with information from historical records.

In the first of these cases (case 5), Stieve gives the length of imprisonment as 50% longer than it was historically, even though he had access to the actual dates of imprisonment and of death in this case (case 5). This can either be a crude miscalculation or an ‘adaptation’ to the histological finding—a marked atrophy of the organs allegedly caused by a long period of “nervous agitation”.

In the four other cases (cases 6–9), the true biographical story—imprisonment, death sentence, and execution of the woman—is replaced by a story that may be constructed to explain some of the histological findings like the degeneration of an oocyte in a developing follicle or the lack of development of a corpus luteum from a ruptured follicle. The wrong circumstances of death given in these cases (suicide, air raids, or execution for murder), meant to conceal the political circumstances, would not affect the scientific argument. However, in all four cases, a preceding stressful event that is obviously invented (another air raid, the death of the fiancé or the husband, denied petition for mercy) is perfectly timed to explain an abnormal morphological development triggered by the influence of the nervous system. We cannot fully exclude that comparable stressful events did occur at the reported time, but were replaced by invented events to ensure anonymity. It nevertheless seems unlikely that Stieve had detailed information about the exact timing of events during the last 2 to 3 weeks of these imprisoned women.

#### Stieve’s possible sources of clinical-biographical information

In the case of specimens from surgical operations, Stieve was either given clinical information by cooperating surgeons [23, p. 62] or had direct access to medical documents [24, p. 26]. As only in one of these cases a named woman could be identified, we did not attempt to search individual medical records. Nevertheless, in general, case sheets of the time did include information as referred to by Stieve like menarche, menstrual cycle or intercourse,^4^ and it seems plausible that Stieve was given access to such records. In the following, we will discuss whether clinical information on women *executed in Plötzensee* may have been as easily accessible for Stieve at the time.

Stieve did have access to court records in at least three cases [8] and took information from them for his case description at least in the one case described above. However, as no further evidence for actual access to records was found, Stieve’s use of such records was likely limited. We suppose that he quickly saw that court records were of no medical value to him. We have scarcely found medical information in the many court records we investigated, and certainly no mention of menstrual bleeding.

To understand whether or not Stieve could gain access to more intimate clinical information from other sources, it is important to understand the processes surrounding the imprisonment and the execution at the time. After their verdict, most women awaited their execution at the women’s prison in Barnimstraße, about 9 km from the execution site at Plötzensee prison (where only men were imprisoned). However, a few women were transported to Plötzensee from other prisons [25], and some only stayed at Barnimstraße for some days or even only for 1 day [26]. The prison was only informed of who was to be executed with very short notice, usually just 1 day beforehand, and the women only learnt of the impending execution when they were taken from their cells to the car bringing them to Plötzensee, where the execution was scheduled for the same day. The transports were accompanied by female prison warders from Barnimstraße.

Stieve mentions doctors and prison warders as his sources of information. As for doctors: In Plötzensee, a medical doctor had to attend the execution, but will not have had regular contact with the women during the short time before their execution. The Barnimstraße prison did not have a prison doctor of its own, external doctors held surgery about twice a week, but medical attention to prisoners was low. Surviving prison records hold very little medical information: we only found very short notes attesting to fitness for work and/or absence of pregnancy.^5^ Moreover, these doctors did not know in advance who would be executed, so that they would have needed meticulous records on all women to be able to give Stieve information, and they certainly had no information on events like the last menstrual bleeding or even “shock bleeding” shortly before the execution.

As for prison warders, they would obviously know about the women’s need for sanitary towels and thus about menstruation. However, the prison had around 100 prison warders, all female, who often changed positions and would therefore not have been able to observe individual women for longer periods of time [26]. It cannot be excluded that the anatomy diener waiting for the bodies in Plötzensee could, as Stieve reported,^6^ ask warders for information when they accompanied prisoners to Plötzensee, or that some warders, given their low salary, could be bribed into relating information from their prison routine. But given the processes described above, this may have been realised in some rare instances, but was hardly a way of producing reliable information to any scientific or medical standard. Also, no indication of such exchanges has ever materialised in any diaries, notes, or records of the time [3].

Finally, it is at least feasible that Stieve obtained information on the length of imprisonment from “*Gefängnisbeamte* [prison officials]”.^7^ Of Stieve’s dissection protocols, 70% do include this information [8]. This is a surprisingly high number, as this information was not part of the official announcements sent to the anatomical institute. On the other hand, only 5 of the 207 protocols include information on the day of the menstrual cycle, which supports the assumption made above that this information was not readily accessible to Stieve. It seems unlikely that he would record the length of imprisonment on the dissection protocols, but not the day of the menstrual cycle, if he knew it.

## Discussion

We examined Hermann Stieve’s published case descriptions, particularly those that can be related to women executed in Berlin Plötzensee during the times of National Socialism. We were able to connect 88 such case descriptions with 33 identified women in an attempt to fact-check the reported information and found a substantial number of instances of reporting wrong or confusing data that need explanation and interpretation.

### Causes of death and/or verdicts

As for the reported sources of tissue and causes of death of the women in question, Stieve did not generally conceal that the tissues and organs he examined came—in part—from execution victims. However, our findings confirm that he concealed the true reasons for the punishment of the victims and in several cases simply invented other causes of death or other verdicts, particularly after 1945 [3, 8]. In publications before 1945, missing or vague information may be partly attributable to an obligation of secrecy surrounding death penalties. After 1945, however, vague or wrong information was clearly a response to accusations of using political victims for his research. Stieve was not so much accused of using the bodies of executed individuals as such—this was not seen as a Nazi-typical practise and did actually continue after 1945 [8]—but that he had used *political* victims and had thus treated them *like common criminals*. The new authorities threatened to bar publications if they were based on research using political victims [3, 5].

While in letters to the authorities, Stieve claimed to have distinguished between political and non-political victims before 1945, he obviously knew that this line was difficult to draw. Thus, if he wanted to continue using his “material” from executed women gained during the war years, he had to conceal their true verdicts in virtually all cases except for two cases of murder. He had no reservations to do so and developed quite some fantasy to construct plausible stories. In the foreword of his 1952 book, he simply claimed that he had only used the bodies of “*Schwerverbrecher* (felons)” [12]—his own reputation was obviously more important to him than the reputation of Nazi victims.

### Clinical information

Even if it remains difficult to fact-check all information that Stieve gives in his case descriptions, particularly information on the menstrual cycle, we have shown that in a relevant number of cases, some of Stieve’s clinical information was either confusing or wrong. On the face of it, we see two possible extremes of explanation along a wide spectrum between “sloppy science” and scientific fraud [27].

On the one side of this spectrum, most of Stieve’s disputable research data can be seen as a consequence of repeated mix-up of individual cases or bits of information, a confusion supported perhaps by hasty or negligent documentation in some cases. This may have been worsened by loss of documentation through war effects—we know that two bombings affected Stieve’s institute and one his home and that these did destroy documents [8] and histological slides [12, p. 167]. Moreover, even during the writing of articles, Stieve constantly incorporated new cases, as he reported in a publication [28] and in a letter to his son.^8^ This approach may have added to a certain chaos, with the support of a scientific argument being more pressing than thorough documentation.

On the other side of this spectrum, some of the reported data may be seen as misleading or even fraudulent. In some of the cases, Stieve’s specifications can be interpreted as being constructed to match the histological findings and to better support the scientific argument. The absence of data on the last menstrual cycle in most dissection protocols may suggest that Stieve simply did not have this data. It cannot be excluded that Stieve was *occasionally* given such information, but the processes surrounding imprisonment and execution of these women make it unlikely that this was on a big scale and in any systematic manner.

It is difficult to decide between these two extremes—or possibly a mix of both—as we have to point to some limitations. First, the number of case descriptions from Stieve’s publications with unequivocally wrong information remains limited. Secondly, the available records of Stieve’s research documentation remain incomplete [8]. Also, of what must have been a big research archive of histological slides, only 172 slides survived [16]—a comprehensive analysis of Stieve’s research approach, as has recently been conducted on Karl Lennert’s histopathological archive [29], is therefore impossible. Thirdly, it cannot be excluded that some of the confusing or misleading clinical-biographical data was part of the effort to conceal the identity of political victims as mentioned above. Evidence thus remains circumstantial and leaves room for interpretation. It must also be cautioned that Stieve’s research methods cannot simply be judged from today’s standards. His case-based approach was more common at the time—systematic research approaches like today’s randomised controlled trials or metaanalyses were all developed after Stieve’s death. There was also no peer review process yet [30].

What remains is a substantial doubt regarding Stieve’s sources for intimate information on the executed women. This doubt has led some authors to suggest that Stieve himself had interviewed or even examined these women at the prison before their death [31, 32] or that Stieve could even “order” executions according to the day of the menstrual cycle [33]. Both these suggestions are very unlikely given the highly regulated judiciary processes surrounding the death penalty in Plötzensee even up to 1945 [2, 3]. Is fraud therefore the only remaining alternative?

Reading Stieve’s publications, a third explanation emerges. It appears that, based on decades of histological experience, Stieve was convinced that he could see the dynamics of the menstrual cycle in his histological slides. From the histological aspect of an ovary, for example, he deduced that a certain follicle would have ruptured either on the same day of death [34, p.555] or “*am nächsten, allerspätestens am übernächsten Tage* [the next day or, at the very latest, the day after]” [35, p.905] or “*in ganz kurzer Zeit* [shortly]” [24, p.37]. Obviously, he was also convinced that he could “see” the day of the menstrual cycle or recent uterine bleeding in his specimens.

From today’s research perspective, we may read into Stieve’s publications a distinction between clinical and histological data that was not that important to Stieve. In many cases, he may simply have judged “clinical” details—like the day of the menstrual cycle—from his histological findings and reported them as “clinical” facts. Stieve was embedded in a research tradition of gynaecological histopathologists like Robert Schröder or Robert Meyer [13] (Schröder is actually the most cited author in Stieve’s 1952 book [12]). For this "thought collective" [36, 37], the menstrual cycle was not so much what a woman reported (or even felt), but rather what could be seen in her tissues. For this group, in which Stieve was so highly esteemed [11], the truth of the menstrual cycle was in the histology rather than in menstrual cycle charts. It is no coincidence that Stieve’s publications never included such charts (as Hermann Knaus requested [38]), but often just the scant indication of a “regular cycle”.

Knaus’ critique questioned the very foundations of this group, which may explain why debates between Stieve and Knaus were so fierce and often emotional [13, 14]. Knaus precisely blamed Stieve for the overestimation of his histological findings. He doubted Stieve’s predictions of ovulations and his calculations of the age of corpora lutea because these were merely based on one single histologically frozen moment in time [38, 39].

From today’s point of view, Stieve was right in doubting the predictability of ovulation suggested by Knaus, but for the wrong reasons: the great natural variability of the menstrual cycle and of the fertility window is attributed to many factors today but not to extracyclical ovulations or direct nervous influence on the ovary [40]. It took until the 1960s that the concept of neurosecretion and of control loops between hypothalamus, pituitary gland, and ovary eventually integrated nervous and endocrine control and that many of Stieve’s positions were refuted, particularly regarding the direct influence of the autonomic nervous system and his “paracyclical ovulations” [14, 41, 42].

## Conclusion

Even if thus many of Stieve’s research results did not stand the test of time, we do not believe that he wilfully and consciously manipulated his data. From today’s point of view, he may have sometimes ‘over-interpreted the facts’ in his analysis of histological images to stay within the "thought style" of himself and his peers—if there is such a thing as pure ‘facts’ in histology, where preconceptions heavily influence the interpretation of an image. This may have been helped by Stieve’s character. He certainly had a tendency to state his findings in an apodictic way. He regularly made statements like “*an dieser Tatsache kann nicht gezweifelt werden* [this fact cannot be doubted]” [22, p.13], and as much as his own findings were “*einwandfrei* [unobjectionable]” [12, 34], he sometimes harshly dismissed others’ findings as “unscientific” [43]. The correspondence of Heinrich von Eggeling (1869–1954), the secretary of the *Anatomische Gesellschaft* at his time, is full of very critical comments by many colleagues,^9^ and von Eggeling himself writes that Stieve should learn “*sich etwas massvoller zu äussern* [to speak out more temperately]”.^10^ This character may not just explain his trouble with colleagues [44], but may also have lacked the flexibility to be open for other interpretations of given data. Stubbornness, however, is not fraud.

This is not meant to defend Stieve from justified accusations. The source of tissues for his research remains ethically dubious, and it remains deplorable that Stieve depicted political victims as “felons” simply to continue publishing. Does it nevertheless matter how much chaotic documentation or an over-interpretation of data influenced some of Stieve’s scientific results? The degree of this influence remains difficult to judge, but if it was significant, this also has consequences for Stieve’s own ethical defence. After 1945 [10, 12], Stieve wrote that all these cases of execution were deplorable, but that he had at least been able to draw scientific insights from them, producing knowledge which “*der ganzen Menschheit Nutzen bringen* [carries a benefit for all of mankind]” [12, p.III]. This utilitarian ethical argument, balancing harm to some individuals against benefit for the majority, is a tricky one in general, but it loses any power if the produced scientific knowledge does not entail this “benefit for all of mankind”.

## Supplementary Information

Below is the link to the electronic supplementary material.

Supplementary file1 (DOCX 194 kb)

## Abbreviations

GDW: *Gedenkstätte Deutscher Widerstand* (German Resistance Memorial Center)
NS: National Socialist
BLHA: *Brandenburgisches Landeshauptarchiv* (State Archive of Brandenburg)
BArch: *Bundesarchiv* (Federal Archive)

## Footnotes

1 Prisoner’s record from the Barnimstraße prison. Landesarchiv Berlin (State Archive Berlin), A Rep. 365, Nr. 142.

2 Files at GDW Berlin: GDW/P StA.Chbg.Reg.Nr.2129/44.

3 Court file, BLHA Potsdam: Rep 12c Berlin II 6296.

4 Krankenblätter (medical case sheets), University Archive Würzburg: UAWü, Op. St. VII 1934, Fall (case) 647, Jahr (year) 1934; Op. St. VII 1939, Fall 206, Jahr 1939.

5 Prisoner’s record from the Barnimstraße prison, *Landesarchiv Berlin* (State Archive Berlin): A Rep. 365, Nr. 98.

6 Letter of 28 November 1946 to the German Administration of Education, University Archive of Humboldt University, Berlin: HUUA, PA St. 116 Bd VI, Bl. 25/26.

7 Letter of 16 January 1946 to Alexander Molzahn, Frankfurt/Main, Archive of former East German Secret Service (BStU), signature: MfS HA IX/11, FV 98/66, Bd. 10, pp. 35–36.

8 Letter of 18 April 1943 to Fritz Stieve (at the time soldier in Africa), University Archive of Humboldt University, Berlin: HUUA, Nachlass Stieve, letter P11.

9 see correspondence with Benninghoff, Bauer, Bluntschli, Elze, Kopsch, Rolshoven, Stöhr, *Nachlass* (estate) von Eggeling, Archive of the Anatomische Gesellschaft, Institute of Anatomy, Erlangen.

10 Letter of 4 August 1934 by von Eggeling to Mollier, *Nachlass* (estate) von Eggeling, Archive of the Anatomische Gesellschaft, Institute of Anatomy, Erlangen, folder 11.
