# Supplementary material for: Anatomist Hermann Stieve used Nazi victims for his research on the menstrual cycle, but did he also fabricate facts?
Source: Arch Gynecol Obstet. 2022 Aug 8;306(4):1287–98. doi: 10.1007/s00404-022-06664-4 (PMC9470644; doi:10.1007/s00404-022-06664-4)
Supplement: Supplementary file 1 — Supplementary file1 (DOCX 194 kb) [file 404_2022_6664_MOESM1_ESM.docx]

**CASES [Supplementary Material]**

**Case 1**

In a publication of 1943, Stieve presents the case of a 38-year-old woman who was married for twelve years and had two children who had "died suddenly on the fourth day after the onset of the last period" [8]. He does not give further clinical or biographical detail and uses a specimen from this woman to demonstrate a normally developed ovarian follicle which is pictured in several figures. The same case is also published in 1952 with a picture of an oocyte, but without further information other than "right ovary of a 38-year-old healthy woman" [2, p138].^[[1]](#footnote-1)^

With a name inscribed on three Neresheimer drawings identical with the published figures, she can be identified as a 38-year-old woman executed in 1942 for high treason. In contrast to Stieve's published information, she was married for eight years and had no children [10, p.258] .^[[2]](#footnote-2)^

It can be argued that Stieve wanted to support the presentation of a normal oocyte in a normally developed follicle by giving the impression that the woman had healthy sexual organs with normal reproductive capabilities. That he knew better is suggested by the fact that the dissection protocol, which does not include biographical information on the number of children, nevertheless explicitly states "0-para [nullipara]", i.e. the dissection findings themselves indicated that the woman had never been pregnant.

**Case 2**

In a publication of 1943, Stieve describes a 30-year-old "entirely healthy, well nourished" woman who had menstruated regularly from age 12, had been married for seven years with three children and had died suddenly on the 14th day after onset of the last period [8]. Stieve stresses that he found "*auch am übrigen Körper nicht die geringste Spur irgendeiner Krankheit* [not the faintest trace of any illness on the entire body]" [8]. Stieve shows images of a freshly ruptured follicle and thus uses the case to present a normal sequence of events with a follicle that ruptured on the day of the menstrual cycle that would regularly be expected. The dissection protocol is rather short and mentions that the dissection was not performed by Stieve himself, but by his colleague Curt Fahrenholz [1].

By a name on one of the drawings and the congruence of information on the named dissection protocol and the published information, the case can be connected with high certainty to a 30-year-old woman who was executed for high treason in 1942 [7, p.216-217]. She had been married since 1938, i.e. not for seven years, and had no children. Notably, she attempted suicide by jumping from a window shortly after being arrested, causing such critical injuries that she actually had to be carried to her trial two months later and to her execution another month later as she could not walk by herself [7, 11].

It is remarkable that Stieve would describe this woman as "entirely healthy". This description appears to be helpful for him to support the portrayal of a normal ovulation in a healthy woman, even though it would have rather been a case in which he should have expected effects of stress. It is difficult to believe that the woman's injuries would not have been visible upon dissection, but we don't know the exact nature of these injuries and it is possible that this was not passed on by Fahrenholz who may have focussed his report on the genital organs. The addition of children to the case description parallels case 1.

**Case 3**

This case has been used four times by Stieve between 1950 and 1952 [2, 5, 9, 12] and also once in 1944 [13]. Stieve describes a 37-year-old "*vollkommen gesunde, geschlechtstüchtige Frau* [perfectly healthy, sexually capable woman]" [2], married since age 23 with four children, who had menstruated regularly about every 28 days and had no medical history except for an appendectomy at age 20. While in 1944, Stieve just speaks of "sudden death", in 1952 he gives an air raid as the cause of death. The day of the menstrual cycle on the day of death is given inconsistently as "middle of the fourth week" in 1944, as "25th day" in 1950, and as "26th day" in 1952. The dissection protocol mentions among others an appendectomy scar, the absence of "striae" (stretch marks), and includes an addition in shorthand "4 children, married since 23rd year".

In his histological findings, Stieve describes an endometrial mucosa in the secretory phase that would fit the 23rd to 27th day of the cycle, a "normal" corpus luteum with an age of about 12-14 days, i.e. compatible with a regular ovulation around the 14th day of the cycle, but also another corpus luteum that shows an age of only two to three days. Stieve concludes from these findings that an additional "paracyclical" ovulation must have occurred two to three days before death.

By her age, the initial Stieve uses in his publications, her name on two Neresheimer drawings and the congruence of the published information with that on the named dissection protocol, the 37-year-old woman can be identified as somebody executed in 1944 for making snide remarks about Hitler.^[[3]](#footnote-3)^ In contrast to the published case description, she was married since age 33 without children,^[[4]](#footnote-4)^ not since age 23 with four children. As, apart from the age, none of the given biographical information is correct – the appendicitis at age 20 may simply have been inferred from the appendectomy scar noted during dissection – the story may give the impression of being freely constructed to support Stieve's concept of an extracyclical ovulation in a woman with otherwise normally functioning genital organs. An alternative explanation would be a mix-up of cases. It is remarkable that Stieve added "4 children" to the dissection protocol at some point, while the protocol itself actually states the absence of stretch marks, suggesting that the woman had never been pregnant.

**Case 4**

In three publications of 1951 and 1952, Stieve tells the story of a 32-year-old woman who had married at age 18, had three children and had lived in adverse circumstances during her last year as her husband had died in action and she had been separated from her children [2, 6, 14]. She allegedly died during an air raid. Stieve uses his histological findings of atrophied ovaries and atretic follicles, which he compares to ovaries of postmenopausal women, to support his concept of a strong influence of the stress situation on ovarian morphology and function via the nervous system in an otherwise "ovary-stable" woman.

By her name inscribed on two Neresheimer drawings, the initial Stieve uses in one publication, and a named dissection protocol, the woman can be identified with high certainty as a 31-year-old woman who was executed for high treason and support of communist resistance in 1943.^[[5]](#footnote-5)^ Apparently, Stieve gets the age wrong by one year because his dissection protocol notes the right year but the wrong month of birth. There is no indication in the historical records that this woman had children.^[[6]](#footnote-6)^ While the story of death during an air raid may be meant to conceal the true cause of death and does not change the scientific conclusions, the mention of three children is obviously meant to support the idea that the influence of the nervous system can be so strong as to change morphology even in previously healthy genital organs.

**Case 5**

This is the case description quoted in section 3.1 as an example for Stieve's reports. In short, Stieve describes a 31-year-old unmarried woman with a regular menstrual cycle since age 14 who was imprisoned for a dangerous crime. After 128 days in prison with amenorrhoea, she received an agitating message (likely the announcement of the imminent execution), suffered a genital bleeding and died eight hours later. Virtually identical versions of this description were published in 1943 [3] and 1952 [2]. In his histological findings, Stieve describes a marked atrophy of the ovarian follicles and of the endometrial mucosa, which he compares to the endometrium of an old woman and which he attributes to the long period of 128 days of "*nervöse Erregung* [nervous agitation]". This atrophied endometrium shows nevertheless signs of a massive bleeding comparable to the loss of upper mucosal layers in regular menstrual bleeding, which however cannot have been triggered by the ovary, but must, in Stieve's view, be the consequence of a direct influence of the nervous system on the uterus.

By the same age and the initial that Stieve uses in his 1952 publication, by her name inscribed on two Neresheimer drawings and by identical information on the named dissection protocol in the published findings, this woman can be identified with a woman who was brought to trial and executed in 1942 for stealing from the property of soldiers who had died in action.^[[7]](#footnote-7)^ The identification seems very certain, even if the same name can also be found, perhaps erroneously, on another drawing that is obviously not related to this case. The case is special in that Stieve had access to court records, which were sent to him three months after her death upon his request^[[8]](#footnote-8)^. The case description includes details that Stieve must clearly have taken from these records, as he partly uses similar wording. This includes the fact that she was an unmarried woman engaged twice and that she worked "diligently and reliably".^[[9]](#footnote-9)^ These records also give the exact day of imprisonment,^[[10]](#footnote-10)^ which Stieve actually copied into his dissection protocol. Between this date of imprisonment and the known date of death, however, there were 83 days, while Stieve nevertheless reports 128 days of imprisonment. This may suggest that Stieve 'adapted' the biographical facts to his histological findings and to his scientific argument by overstating the duration of imprisonment. The only alternative explanation would be a gross miscalculation of the number of days between the two given dates. The dissection protocol includes several columns of figures next to the note "128 days", but they are rather confusing and do not explain how Stieve calculated this number. On another dissection protocol, such columns of figures do actually represent the days of each month between imprisonment and death, but Stieve forgot one month when calculating (figure 1).

**Fig. 1** Detail of dissection protocol no. 5 for a 22 year-old woman who was imprisoned from mid-September until her execution on 15 April 1943 and for whom Stieve reported 181 days of amenorrhoea [2, p. 64]. Stieve obviously used this column of figures to calculate the number of days, but misses either December or January. The note on the left "*wichtig, gezeichnet*" translates as "important, drawn".

**
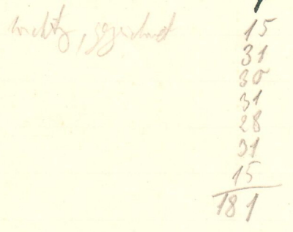
**

**Case 6**

This case is mentioned in section 3.2.1 as the case of an alleged suicide. In three publications of 1950 to 1952 [2, 5, 9], Stieve describes a 25-year-old woman who had been engaged for two years but since eight months was separated from her fiancé who fought in the army. She had kept a menstrual calendar and had had a phase of amenorrhoea after the separation from her fiancé, but for the last five months had resumed regular bleeding. 15 days after her last menstrual bleeding, the woman learnt of the death of her fiancé and another 9 days later, i.e. on the 25th day of her cycle, she committed suicide [2, p.40]. Stieve's scientific argument in this case concerns a near-mature follicle in one of the ovaries that according to him would have ruptured around the 16th to 18th day of the menstrual cycle, but holds an oocyte with signs of degeneration. Stieve attributes this degeneration to "*starke Erregung* [strong agitation]" on the 15th day of the cycle [2, p.43].

The age, the initial used in Stieve's 1952 publication, the name on four Neresheimer drawings and the congruent information on the named dissection protocol identify this woman as a 25-year-old who was executed for alleged espionage in 1943 [4]. According to historical sources, she was in prison for 10 months and sentenced to death more than three months before her death [4]. We ignore whether the woman was engaged and/or lost her fiancé shortly before her death, but given the true circumstances of her death, the story may be seen as constructed. Notably, the assumed time of death of the fiancé perfectly fits the assumed time of the degeneration of the oocyte as visible in the histological specimens.

**Case 7**

In his 1952 book, Stieve describes the case of a 21 year-old woman and reports: "The last menstruation occurred in February, then the woman's home was destroyed by bombs, her husband lost his life. From this moment the woman lived in rather adverse circumstances, which agitated her a lot. About 14 days after the start of her last bleeding, she lost her life in another bombing." [2] Very similar to case 6, Stieve sees the nervous agitation around 14 days before death as an explanation for signs of degeneration in an oocyte in a "*fast sprungreifer* [nearly mature]" ovarian follicle.

The age, the published initial, the name on one of the Neresheimer drawings and the named dissection protocol allow a safe identification of the woman behind this case. The 21-year-old woman was part of a resistance group and was sentenced to death for high treason and executed in 1943.^[[11]](#footnote-11)^ She was in prison for nine months [11], her husband, who was part of the same resistance group, was executed six months before her^[[12]](#footnote-12)^ while it remains unclear when she learnt about her husband's death [7, p215-217]. Stieve thus implies a stressful period of only about 14 days, while the true historical events would have spoken for many stressful months before this woman's death. As in case 6, the invented stressful events match the histological findings better than the historical events.

**Case 8**

In his 1952 book, Stieve also tells a similar story about a 23-year-old unmarried woman with no history of disease who suffered the destruction of her home through bombing on the 10th day of her menstrual cycle. She barely survived, but died 15 days later, i.e. on the 25th day of her cycle, "due to external forces" [2]. The same case is decribed with less detail in 1950, but giving the 26th day of the menstrual cycle as the day of death [9]. Following the dissection protocol, which lists the (alleged?) exact date of the last menstruation and of death, it would actually have been the 24th day of the cycle. Stieve's scientific argument in this case is complicated, he explains that the endometrium would rather match an earlier period around the 14th day of the cycle and that one ovary includes a mature follicle that would have ruptured "likely in 1-2 days", i.e. around the 28th day of the cycle. The other ovary, however, holds a collapsed follicle that according to Stieve must have ruptured around a week ago, but, due to nervous agitation, did not produce a corpus luteum.

The age, published initial, the name on four Neresheimer drawings and the match of details between the dissection protocol and the published case allow the safe identification of a 23-year-old woman who was actually arrested for plundering after an air raid three months before her death and sentenced to death 18 days before the execution in 1944.^[[13]](#footnote-13)^ In contrast to most other cases, this woman's court records do include medical data on two recent hospitalisations for angina and for oophoritis,^[[14]](#footnote-14)^ which stands against the absence of previous disease stated by Stieve. As his claim that the woman was unmarried is also wrong,^[[15]](#footnote-15)^ it seems that Stieve did not have access to much information, and neither is there evidence that he knew the historical dates of her imprisonment and trial. Therefore, the story of the stressful last 15 days of her life may have been constructed not only to obscure the true cause of death, but also to explain the failure of corpus luteum formation around one week before death. If Stieve knew of the trial 18 days before her death, he may have replaced this stressful event with the invented story of bombing 15 days before death – which actually fits the histological findings even better. The dissection protocol (see Fig. 2) shows two later additions (both in blue instead of black ink): the words "*reifender Follikel* [maturing follicle]" added to the histological description and the note "*letzte Regel 27.9.44* [last bleeding 27 September 44]" in the upper right-hand corner. As this exact date is the only detailed information in this case, it may well have been 'constructed' to match the mature follicle and added here for use in later publications.

**Fig. 2** Details of the dissection protocol for the 23-year-old woman described in the text (unnumbered protocol, filed before no. 1). Additions in blue ink. The left detail reads "*letzte Regel* [last period] *27.9.44*", the right one "= *reifender Follikel* [maturing follicle]".


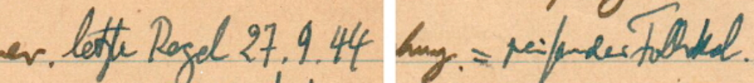


**Case 9**

In four publications of 1950 and 1952, Stieve describes the case of a 35-year-old woman who was married and had two children, the last was born when she was 28. After the death of her husband she "led a furious life and consorted with various men" and poisoned seven men by arsenic. She was in prison for 90 days, where she menstruated three times. 14 days before her death, she learnt that her petition for mercy had been denied and became "extremely agitated and restless". She was executed on the 29th day after her last period [2, p.14]. Stieve's scientific argument in this case is that a follicle that according to its histological appearance must have ruptured on the 14th to 18th day of the cycle, did not develop into a corpus luteum because of nervous agitation in this period of time.

By the published age and initial, the name on two Neresheimer drawings and details from the named dissection protocol, she can be identified as a 35-year-old woman who was arrested and executed in February 1945 for plundering after an air raid. The historical records confirm that she had two children, although the last one was born when she was 23 years old, not 28. ^[[16]](#footnote-16)^ She was sentenced to death about 50 days before her execution, the exact duration of imprisonment is not known, and neither do we know whether and when there was a petition for mercy. As has been noted in section 3.2, the case of the sevenfold murder by poisoning does actually describe another woman executed in 1944.

It is striking that the timing of the reported stressful event, in this case the denied petition for mercy, falls exactly into the time period when according to the histological findings Stieve assumes the missed development of a corpus luteum due to nervous stress. This lends itself to the assumption that this stressful event was 'constructed' to match the histological findings, but we do not have archival sources to prove Stieve wrong in this.

**References**

1. Winkelmann A. The "economy of truth" - New historical sources allow new insights into Hermann Stieve's use of the bodies of execution victims for research. Ann Anat 2022. DOI: 10.1016/j.aanat.2022.151893
2. Stieve H. Der Einfluß des Nervensystems auf Bau und Tätigkeit der Geschlechtsorgane des Menschen. Stuttgart: Thieme; 1952.
3. Stieve H. Schreckblutungen aus der Gebärmutterschleimhaut. Zentralbl Gynäkol 1943; 67: 866-877.
4. Zawacka E. Monika Dymska (1918-1943). In: Scientific Society of Gdansk, ed. Zasluzeni Pomorzanie w latach II wojny swiatowej : szkice biograficzne [Meritous Pomeranians during World War II: biographical sketches]. Wroclaw: Ossolineum; 1984: 60-64.
5. Stieve H. Angeblich sterile Zeiten im Leben geschlechtstüchtiger Frauen. Z Geburtsh Gynäkol 1952; 136: 117-136.
6. Stieve H. Die Geschlechtsorgane der alternden Frau und die Bedeutung der Altersveränderungen für die Entstehung von Mißbildungen. Verh Anat Ges 1951; 1951: 23-48.
7. von Gélieu C. Barnimstraße 10. Das Berliner Frauengefängnis 1868-1974. Berlin: Metropol Verlag; 2014.
8. Stieve H. Über Follikelreifung, Gelbkörperbildung und den Zeitpunkt der Befruchtung beim Menschen. Z Mikrosk Anat Forsch 1943; 53: 467-582.
9. Stieve H. Die heutige Auffassung über den Ablauf des Zyklus und seine Anomalien. Zentralbl Gynäkol 1950; 72: 897-907.
10. Coppi H, Danyel J, Tuchel J. Die Rote Kapelle im Widerstand gegen den Nationalsozialismus. Berlin: Hentrich / Gedenkstätte Deutscher Widerstand; 1994.
11. Atzili A. Baum Gruppe: Jewish Women. (Shalvi/Hyman Encyclopedia of Jewish Women). 1999. DOI: https://jwa.org/encyclopedia/article/baum-gruppe-jewish-women
12. Stieve H. Cyclus, Physiologie und Pathologie (Anatomie). Arch Gynäkol 1952; 183: 178-203.
13. Stieve H. Paracyclische Ovulationen. Zentralbl Gynäkol 1944; 68: 257-272.
14. Stieve H. Die Oozytenschwäche der alternden Frau. Zentralbl Gynäkol 1951; 73: 637-643.

1. p138 [↑](#footnote-ref-1)
2. See also files at the archive of the GDW Berlin. [↑](#footnote-ref-2)
3. Arrest warrant of 24 October 1943, BArch Berlin: R3017/32940 p. 11. [↑](#footnote-ref-3)
4. Interrogation protocol of 25 September 1943 by *Geheime Staatspolizei* (Secret State Police), BArch Berlin: R3017/32940 p. 6-7. [↑](#footnote-ref-4)
5. *Strafprozessakte* (criminal case file), BArch Berlin: R 3018/1612, volumes 1, 4, 5, 7. [↑](#footnote-ref-5)
6. *Strafprozessakte* (criminal case file), BArch Berlin: R 3018/1612, volumes 1, 4, 5, 7. [↑](#footnote-ref-6)
7. Court file, BLHA Potsdam: Rep 12c Berlin II 6296. [↑](#footnote-ref-7)
8. Letter of 12 October 1942 from Hermann Stieve to the archive of the prosecution office of the *Landgericht* (district court) Berlin, BLHA Potsdam: Rep. 12c Berlin II 6296 p. 105. [↑](#footnote-ref-8)
9. Proclamation of sentence of 6 June 1942, BLHA Potsdam: Rep. 12c Berlin II 6296 p. 80. [↑](#footnote-ref-9)
10. *Gefangenenkarte* (Prisoner file card) of 20 April 1942, BLHA Potsdam: Rep. 12c Berlin II 6296 p. 11. [↑](#footnote-ref-10)
11. *Strafprozessakte* (criminal case file), BArch Berlin: R 3018/1642. [↑](#footnote-ref-11)
12. *Strafprozessakte* (criminal case file), BArch Berlin: R 3018/1642. [↑](#footnote-ref-12)
13. Court files, BLHA Potsdam: Rep. 12c 1678. [↑](#footnote-ref-13)
14. Interrogation protocol of 14 May 1944, BLHA Potsdam: Rep. 12c 1678 p. 6. [↑](#footnote-ref-14)
15. Interrogation protocol of 14 May 1944, BLHA Potsdam: Rep. 12c 1678 p. 5. [↑](#footnote-ref-15)
16. Prisoner’s record from the Barnimstraße prison. Landesarchiv Berlin (State Archive Berlin), A Rep. 365, Nr. 142. [↑](#footnote-ref-16)
